# Fractures of the knee in children—what can go wrong? A case file study of closed claims in The Patient Compensation Association covering 16 years

**DOI:** 10.1007/s11832-015-0684-6

**Published:** 2015-09-25

**Authors:** V. Leeberg, S. Sonne-Holm, J. Krogh Christoffersen, C. Wong

**Affiliations:** Department of Orthopaedics Surgery, Hvidovre University Hospital, Kettegaard Alle 30, 2650 Hvidovre, Denmark; The Patient Compensation Association, Nytorv 5, 3., 1450 Copenhagen K, Denmark; Silkeborggade 29, 2. tv, 2100 Copenhagen, Denmark

**Keywords:** Paediatrics, Orthopaedics, Closed claims, Tibial fracture, Knee

## Abstract

**Introduction:**

Intra-articular knee fractures in children are rare. The Patient Compensation Association (PCA) receives claims for financial compensation from patients who believe they have sustained damage from their treatment in the health care system. We used relevant cases of closed claims to identify causality and co-factors contributing to these apparent malpractices.

**Materials and methods:**

A partial root core analysis was performed on closed claims from the PCA database concerning proximal tibial fractures in children aged ≤15 years.

**Results:**

We identified 13 cases. The main complaint was missed diagnosis (6 cases)—fractures of the tibial eminence were the main culprit, with damage to the popliteal artery caused by a medial condyle fracture being the most serious. All cases were missed by junior doctors. Secondary complaints were problems with casting, dissatisfaction with correct treatment, and insufficient surgery or complications relating to surgery. Eight of the complaints were acknowledged, with six receiving financial compensation ranging from EUR 9,600 to EUR 70,000. Five out of the six cases of missed diagnosis were acknowledged.

**Conclusions:**

This study indicates that recognizing the degree of injury to the knee in children, which should include an X-ray examination, is key to preventing missed diagnosis and delayed and potentially more difficult surgery with long-lasting sequelae for the child. The PCA database seems to be a useful way to highlight systematic problems in the Danish health care system and could potentially be an important means to improving patient safety and preventing treatment-related injuries.

## Introduction

Intra-articular knee fractures in children are rare, and injury patterns in children differ from those of adults, who sustain anterior cruciate ligament damage instead of the eminentia avulsion fracture most often seen in children [1–3]. The rarity of this fracture makes it more likely to be missed and patients less likely to receive adequate conservative or surgical treatment. However, the fracture can have serious consequences for the child later in life due to damage to the intra-articular structures with potential instability and secondary arthrosis of the knee. However, this problem has not yet been examined thoroughly by long-term follow-up [2].

The Danish Parliament passed the Patient Insurance Act in July 1992 [4]. This gives patients the possibility to file a claim for financial compensation to The Patient Compensation Association (PCA) (formerly The Danish Patient Insurance Association; DPIA) if they have sustained an injury or unexpected side-effect from a medical action. The PCA is based on a ‘no blame/no fault’ principle and a claim to the PCA will have no legal or disciplinary consequences for the health professionals involved. Filing a claim to the PCA is free of charge for the plaintiff.

The PCA will decide if the injury is actually from the treatment and not for instance an expected side-effect to the disease or trauma itself. Acknowledgement is given under the following circumstances:

1. An experienced specialist would have acted differently, whereby the injury could have been avoided.
2. Defects in or failure of technical equipment were a major factor in the incident that caused the injury.
3. The injury could have been avoided by using alternative treatments, techniques or methods, if these are considered to be equally safe and potentially offer the same benefits.
4. The injury is rare, serious and more extensive than the patient should be expected to endure.

**Fig. 1:**
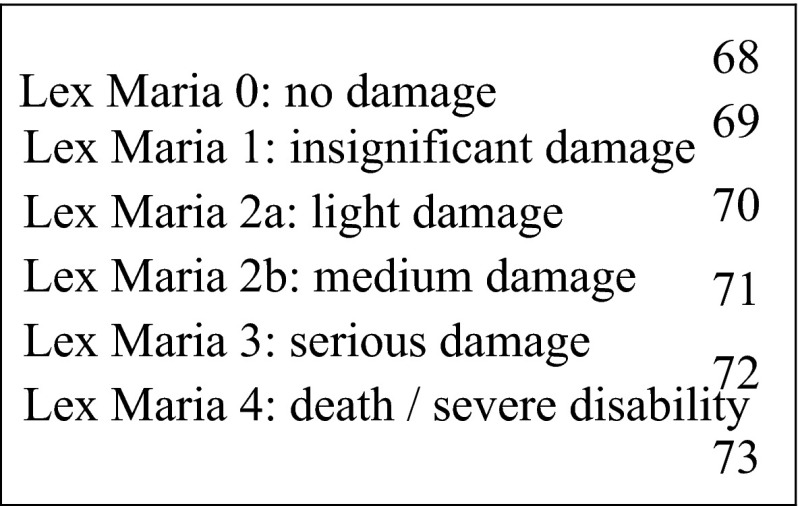
The PCA classifies the degree of injury according to Lex Maria, which was named after a case in Sweden at the Maria Hospital where four patients died after being injected with a disinfectant by mistake

The PCA considers the severity of the injury when calculating the size of financial compensation, taking other factors into consideration such as the amount of pain and suffering, degree of permanent injury, reduced ability to work, reduced income and additional medical expenses. Moreover, the injury is classified according to Lex Maria (Fig. 1). There is a triviality limit of Euros 1,500. A patient can appeal the decision of the PCA to the Patient Damage Appeal Board and to the Danish Court of Law.

To date, the PCA has received >50,000 claims. The approval rate over the past 5 years has been approximately 32 % [5]. These claims and decisions are saved in the PCA database together with medical journals and statements from specialists used as the ruling in the case. This database, which contains cases over the past 16 years, makes it possible to perform closed claim analysis to identify and examine unfortunate patterns in the treatment of specific diseases and possibly in the Danish health care system.

In this study we wanted to identify causality and co-factors contributing to these apparent malpractices and examine the potential health consequences of knee fractures in children by evaluating cases from the PCA.

## Materials and methods

The DPIA database contains medical journals, analyses and statements from medical consultants, internal notes from the PCA, and a written evaluation and the decision for each case. This study is a retrospective systematic review of relevant closed claims in order to identify possible patterns in apparent malpractices. The files of each case were thoroughly and systematically examined. We called it a partial root core analysis because we only looked for reasons for the injuries and not, at the time, any possible solutions to the systematic problems we might find.

The PCA database was searched using the WHO classification of diseases for proximal tibial fractures DS821 including the subdivisions DS821A-D. Only aged patients aged ≤15 years were included. Every case file was thoroughly examined and divided into groups by fracture type and by reason for filing a claim to the PCA.

## Results

A total of 19 cases were found, dating from 1995−2011. Three of the cases were excluded because of wrong encoding (not fractures of the proximal tibia). Three of the patients were registered twice and were analysed as one case per patient, leaving 13 cases (7 male, 6 female) with a mean age of 10.2 years (1–15 years).

The results, which are summarized in Table 1, are described in more detail in the following section.

**Table 1:** Summary of the relevant cases

Age (years)	Gender	Activity	WHO diagnosis	Fracture morphology	Treatment	Complication 1	Treatment of complication
1	M	Fall from approximately 1 m	DS821	Non-dislocated fissure of proksimale tibia 2 cm below the epiphyseal line Infraction of fibula	Casting, circular after 3 days. Total of 4 weeks. Full weight bearing	Genus valgus	Followed by paediatric orthopaedic specialist
3	F	Trampoline	DS821	Non-displaced transverse fracture 1.4 cm distal to the epiphysis	Casting 3 weeks. No weight bearing	Cast below knee instead of above knee	Casting for 3 more weeks. Above knee cast
4	F	Sledging	DS821	Dislocated fracture through the epiphysis and the tibial shaft, non-dislocated fracture of the fibula	Internal fixation with Kirschner-wires. Circular cast above knee 6 weeks	Suboptimal placement of Kirschner wires	Secondary surgery 2 days later
4	M	Trampoline	DS821	Non-dislocated transverse fracture of the tibial shaft 3 cm distal to the joint	Cast above knee 6 weeks	Pes equinus in cast, decubitus of heel	Change of cast
10	F	Skiing	DS821D	Dislocated comminuted fracture of the eminentia	Athroscopic fixation with biofix rods. ROM-splint 0–10° 5 weeks. Full weight bearing	Non-union	Secondary fixation with osteo-suture 7 months after. Brissement force 8 months after
12	F	?	DS821	Epiphysiolysis	None Missed diagnosis		
12	F	Jump from a slope	DS821C	Non-dislocated fracture of both condyles and the eminentia	Circular cast above knee 6 weeks	Continues pain. Limited movement	Diagnostic arthroscopy. Big chondral injury
13	M	Motor cross	DS821D	Dislocated eminentia avulsion	ROM splint 0-10° 1 week Fracture not diagnosed before 7 weeks after injury	None	None
14	M	Team handball	DS821	Dislocated epiphysiolysis	Internal fixation with Kirschner wires	Infection	Synovectomi + hardware removal 10 days post op followed by above knee casting and antibiotics. Synovectomi and brissement forcé 3 months after followed by CPM treatment
14	F	Bicycle	DS821D	Eminentia avulsion with pseudo arthrosis 4 months after injury. Not X-rayed primarily	None Missed diagnosis	Continues pain	Arthroscopic fixation with 2 smart nails 5 months later. 2.5 years later arthroscopic reduction of the eminentia fragment and notch plastic
15	M	Moped	DS821C	Dislocated fracture of the medial condyle	Surgery planed for next day	Lesion of popliteal artery	Fasciotomi suture of the popliteal artery, Fracture treated with 2 cannulated screws
15	F	Mountain bike	DS821D	Eminentia avulsion. Not X-rayed primarily	None Missed diagnosis	Continues pain	Eminentia removed and ACL resected 1 month after when attempt to fixate it failed
15	M	?	DS821B	Small osteochondral lesion after dislocation of the patella	Conservative		Removal of osteochondral fragment

Age (years)	Gender	Complication 2	Reason for complaint	Lex Maria\	Acknowledged	Compensation (Euros)	Follow-up (months)	Charge of doctor involved
1	M		Dissatisfied with correct treatment	1	No. Not injured by the treatment	0	10	?
3	F	Leg 1.2 cm shorter	Casting problems	1	Yes. Prolonged period of illness	Not settled	8	Junior
4	F	Peroneal paralysis. Nerve intact on ENS	Dissatisfied with correct treatment	0	No. Not injured by the treatment	0	26	Consultant
4	M	Reduced dorsal flexion and general reduced muscle strength. Normal function after rehabilitation	Casting problems	1	No. Not injured by the treatment	0	9	Junior
10	F	Limited ability to extend the knee and shortening at the Achilles tendon	Insufficient surgery	2a	Yes	70,000	20	Consultant
12	F		Missed diagnosis	1	No. No damage	0	47	Junior
12	F		Dissatisfied with correct treatment	0	No. Not injured by the treatment	0	54	Junior
13	M	None	Missed diagnosis	2b	Yes. But under triviality limit	0	15	Junior
14	M	Pain, limited movement of knee	Complications. Period of illness prolonged	2b	Yes. Severe complication	30,000	16	Consultant
14	F	Limited extension (normal range of movement after 2nd surgery) Continues pain	Missed diagnosis and insufficient surgery	2a	Yes. Missed diagnosis	9,600	38	Junior
15	M	Partial peroneal paralysis, mild instability of knee. Flexion contracture of 1st toe	Missed diagnosis	3	Yes. Missed diagnosis	24,000	30	Junior
15	F	ACL instability	Missed diagnosis	2a	Yes. Missed diagnosis	Not settled	5	Junior
15	M		Missed diagnosis	1	Yes. But under triviality limit	0	39	Junior

*ACL* Anterior cruciate ligament, *ENS* electric nerve stimulation, *?* unknown

The fracture morphologies were based on the X-ray findings, which were available in eleven cases, and included six dislocated fractures. In the remaining two cases, the journals were only partially available; however, they were not excluded since they were otherwise consistent with the other cases.

Four patients received acute surgical treatment. The rest were treated conservatively or received no treatment; the latter were not treated due to missed initial diagnosis. Seven patients required delayed surgery.

One case had more than one complaint. The main complaint was missed diagnosis—a fracture interpreted as a soft-tissue injury with no radiological examination primarily (6 cases). All cases were missed by junior doctors. Eminentia fractures were the main culprit, with damage to the popliteal artery caused by a medial condyle fracture being the most serious.

Secondary complaints were problems due to inadequate type and duration of casting (2 cases), dissatisfaction with correct treatment (3 cases) and insufficient surgical procedures or complications (3 cases), with infection after internal fixation of a proximal tibia fracture being the most serious.

**Fig. 2:**
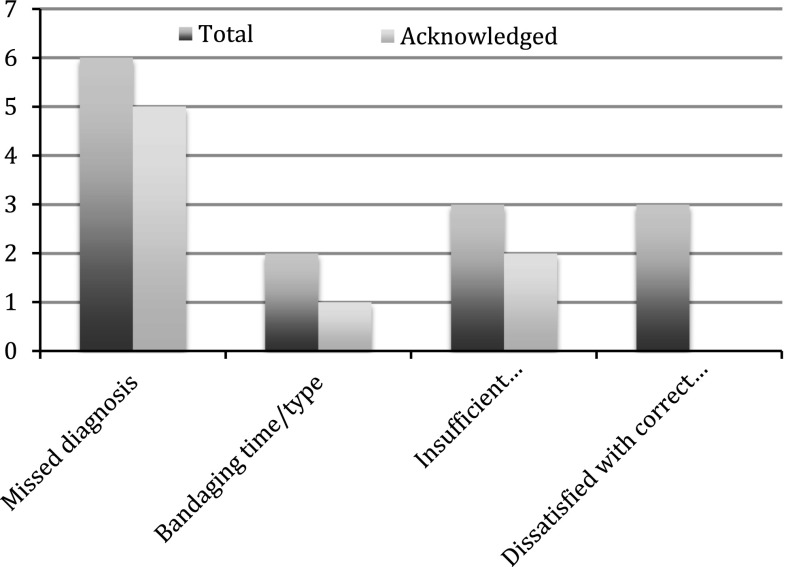
Distribution of complaints and number of acknowledged cases

Of the eight claims acknowledged by the PCA, 6 received financial compensation ranging from EUR 9,600 to EUR 70,000. In two cases, the size of financial compensation was not settled at the time of the review and two cases were below the triviality limit. Five out of the 6 cases of missed diagnosis were acknowledged. In addition to these 5 cases, acknowledgment was also given to one case of postoperative infection, one case of inadequate casting prolonging the treatment period, one case of insufficient surgery and one case of missed diagnosis which also complaint about insufficient surgery. This is illustrated in Fig. 2.

Lex Maria classifications of 2a or higher all lead to acknowledgement; however, the Lex Maria classification has no influence on the size of financial compensation. There was no association between the fracture type and Lex Maria classification.

## Discussion

In this closed claims analysis we examined complaints relating to knee fractures in children. Eight of the 13 cases were acknowledged, which is high compared to the general percentage (32 %) of acknowledgment by the PCA [5]. Claims acknowledged according to the PCA criterion 1 (see introduction) can be classified as malpractice and preventable and claims acknowledged by criteria 2–4 as accidental. Seven of the eight acknowledged cases were acknowledged by criterion 1 and could have been prevented. Initial missed diagnosis was the main complaint in our findings. Fractures were missed because of insufficient evaluation of the patient, i.e., no X-ray examination. Most of the fractures were sustained during play and sports and not due to major trauma, which correlates with the literature [6–9]. Therefore, the fact that the diagnosis is missed cannot be explained by atypical trauma mechanisms or patients presenting with atypical histories, but by a lack of awareness of the disease and insufficient evaluation and examination of the patients.

Delayed diagnosis can have an extensive influence on the child’s later functional level, which is reflected by the high Lex Maria classification and acknowledgment by the PCA. The children in this study, who received correct initial treatment when the fracture was first diagnosed, suffered from and complained of well-known complications (i.e., infection, growth disturbances, nerve damage caused by the fracture). This stresses the importance of correct initial diagnosis and treatment; a study by Pihl et al. showed that delayed diagnosis of paediatric orthopaedic illnesses can be harmful [10].

As our study was based on a small selected cohort, no meaningful statistical analysis could be made. The number of children with similar fractures and negligent treatment is possibly a lot higher. It has been estimated that only 2 % of patients sustaining an injury in relation to treatment actually file a complaint [11].

This study was carried out as part of a group of similar closed claimed analysis studies in the PCA concerning different paediatric fractures and orthopaedic illnesses (ankle fractures, supracondylar humerus fractures, physeal injuries of the distal humerus, developmental hip dysplasia and slipped capital femoral epiphysis). To date, not all of these studies are published [10], but the abstracts are available in the Danish Orthopaedic Society Bulletin for 2013 and 2014 (http://www.ortopaedi.dk). When these studies are combined, there will be a total of 244 cases. They show a strong trend toward missed diagnosis and delayed treatment being a considerable problem in the treatment of children with orthopaedic illnesses. As seen in our group of patients, the delay in treatment is related to patients being seen by junior doctors or doctors with little orthopaedic experience in the acute phase of the injury or at the onset of symptoms. Patient safety could be seen as compromised by the structure of the Danish healthcare system, where young inexperienced doctors work at the front line. To make the system safe, proper training and education is needed for the frontline staff, experienced orthopaedic surgeons should evaluate the quality of the examinations and treatment, and radiologists should review the X-rays. This would be a small socioeconomic investment with a high impact on the quality of treatment and subsequent quality of life for the affected children.

Finally, closed claim analysis is a methodology used to highlight systematic failures and procedural weaknesses [12, 13]. It has been used especially in the field of anaesthesiology [14–16]. To our knowledge, this is the first internationally published study in orthopaedic surgery, where the methodology of closed claims analysis has been applied successfully.

## Conclusion

Enhanced attention to trauma mechanism by educating the initial treating doctors would improve the quality of acute-phase treatment of fractures in the knee in children.

The PCA database seems to be a useful way to enlighten systematic problems in the Danish health care system and could potentially be an important means to improving patient safety and preventing treatment-related injuries.

## References

[1] Meyers MH, McKeever FM (1959). Fracture of the intercondylar eminence of the tibia. J Bone Joint Surg Am.

[2] Leeberg V, Lekdorf J, Wong C, Sonne-Holm S (2014). Tibial eminentia avulsion fracture in children—a systematic review of the current litterature. Dan Med J..

[3] Fehnel DJ, Johnson R (2000). Anterior cruciate injuries in the skeletally immature athletes: a review of treatment outcomes. Sports Med.

[4] http://patienterstatningen.dk/da/Love-og-Regler.aspx. Accessed 18 Sept 2015

[5] http://patienterstatningen.dk/da/Udgivelser-og-tal/Aaret-i-tal.aspx. Accessed 18 Sept 2015

[6] Hunter RE, Willis JA (2004). Arthroscopic fixation of avulsion fractures of the tibial eminence: technique and outcome. Arthroscopy.

[7] Senekovic V, Veselko M (2003). Anterograde arthroscopic fixation of avulsion fractures of the tibial eminence with a cannulated screw: five-year results. Arthroscopy.

[8] Reynders P, Reynders K, Broos P (2002). Pediatric and adolescent tibial eminence fractures: arthroscopic cannulated screw fixation. J Trauma.

[9] Iborra JP, Mazeau P, Louahem D, Diméglio A (1999). Fractures of the intercondylar eminence of the tibia in children. Apropos of 25 cases with a 1–20 year follow up. Rev Chir Orthop Reparatrice Appar Mot.

[10] Pihl M, Sonne-Holm S, Christoffersen JK, Wong C (2014). Doctor’s delay in diagnosis of slipped capital femoral epiphysis. Dan Med J..

[11] Localio AR, Lawthers AG, Brennan TA (1991). Relations between malpractice claims and adverse events due to negligence. Results of the Harvard Medical Practice Study III. N Engl J Med.

[12] Hove LD, Bock J, Christoffersen JK, Andreasson B (2010). Analysis of 136 ureteral injuries in gynaecological and obstetrical surgery from completed indurance claims. Acta Obstet Gynecol Scand.

[13] Hove LD, Bock J, Christoffersen JK (2012). Analysis of deaths among children in the period 1996–2008 from closed claims registered by the Danish Patient Insurance Association. Acta Paediatr.

[14] Cheney FW (2010). The American Society of Anaestesiologists closed claims project: the beginning. Anesthesiology..

[15] Ranum D (2014). Analysis of patient injury based on anesthesiology closed claims data from a major malpractice insurer. J Health Risk Manag.

[16] Dutton RP (2014). Massive haemorrhage: a report from the anesthesia closed claims project. Anesthesiology.

